# Metabolic status differentiates Trp53inp2 function in pressure-overload induced heart failure

**DOI:** 10.3389/fcvm.2023.1226586

**Published:** 2023-12-18

**Authors:** Jianfang Liu, Tian Liu, Shuxun (Vincent) Ren, Cansheng Zhu, Eyad Bouso, Samir Mamlouk, Christoph D. Rau, Yibin Wang, Chen Gao

**Affiliations:** ^1^ Department of Cardiology, Renmin Hospital of Wuhan University, Wuhan, China; ^2^ Department of Pharmacology and Systems Physiology, University of Cincinnati, Cincinnati, OH, United States; ^3^ Signature Research Program in Cardiovascular and Metabolic Diseases, DukeNUS Medical School, Singapore, Singapore; ^4^ Department of Genetics, School of Medicine, University of North Carolina, Chapel Hill, NC, United States

**Keywords:** Trp53inp2, glucose metabolism, cardiometabolic syndrome, transcription factor, heart failure

## Abstract

Cardiometabolic disorders encompass a broad range of cardiovascular complications associated with metabolic dysfunction. These conditions have an increasing share in the health burden worldwide due to worsening endemic of hypertension, obesity, and diabetes. Previous studies have identified Tumor Protein p53-inducible Nuclear Protein 2 (Trp53inp2) as a molecular link between hyperglycemia and cardiac hypertrophy. However, its role in cardiac pathology has never been determined *in vivo*. In this study, we generated a cardiac specific knockout model of Trp53inp2 (Trp53inp2-cKO) and investigated the impact of Trp53inp2 inactivation on the pathogenesis of heart failure under mechanic or/and metabolic stresses. Based on echocardiography assessment, inactivation of Trp53inp2 in heart led to accelerated onset of HFrEF in response to pressure-overload, with significantly reduced ejection fraction and elevated heart failure marker genes comparing to the control mice. In contrast, inactivation of Trp53inp2 ameliorated cardiac dysfunction induced by combined stresses of high fat diet and moderate pressure overload (Cardiometabolic Disorder Model). Moreover, Trp53inp2 inactivation led to reduced expression of glucose metabolism genes in lean, pressure-overloaded hearts. However, the same set of genes were significantly induced in the Trp53inp2-cKO hearts under both mechanical and metabolic stresses. In summary, we have demonstrated for the first time that cardiomyocyte Trp53inp2 has diametrically differential roles in the pathogenesis of heart failure and glucose regulation under mechanical vs. mechanical plus metabolic stresses. This insight suggests that Trp53inp2 may exacerbate the cardiac dysfunction during pressure overload injury but have a protective effect in cardiac diastolic function in cardiometabolic disease.

## Introduction

The cardiometabolic syndrome represents a broad range of cardiovascular diseases associated with metabolic abnormalities, and has become a major public health problem in the United States as well as many countries worldwide (1). The cardiac pathology associated with cardiometabolic syndrome can often be traced to functional and structural alterations in other organ systems, including liver, fat and skeletal muscles (2). Consequently, metabolic disturbances at systemic level, such as hyperglycemia, hyperlipidemia, and insulin resistance have demonstrated causal effects to the pathogenesis of cardiometabolic syndrome based on numerous epidemiological studies (3, 4), as well as extensive preclinical studies involving animal models under simultaneous application of mechanical and metabolic stresses (5, 6). Despite the broad recognition to the importance of metabolic stress in heart failure development, the molecular network linking metabolic disturbance and pathological progression in heart is vastly underexplored.

Heart is one of the most metabolically demanding organs with high level and constant demand for ATP production in order to maintain cardiac contractility. Under normal condition, the adult heart prefers to utilize fatty acid as the main fuel source. However, in failing heart, the glucose becomes a more significant substrate when fatty acid oxidation is impaired (7). Glucose metabolism in heart is tightly regulated by both transporters and metabolic enzymes, and impaired glucose metabolic activities could have profound impact on cardiac contractile function and hypertrophic remodeling (8–10). In the past decade, targeting glucose metabolism has also been demonstrated to be an effective therapeutic strategy to treat heart failure (11, 12).

Trp53inp2 (Transformation related protein 53 inducible nuclear protein 2) is originally discovered as a modulator of thyroid hormone receptor in skeletal muscle as well as a candidate gene associated with obesity in adipose tissues (13, 14). Based on its subcellular localization, Trp53inp2 is postulated to have dual roles in nucleus and cytoplasmic compartments. In the nucleus, Trp53inp2 enhances the transcriptional activity of the thyroid hormone receptor and regulates promoter activities of ribosomal genes, and subsequently promotes ribosome biogenesis (14, 15). In the cytosol, Trp53inp2 regulates autophagosome biogenesis by promoting LC3B-ATG7 interaction and serves as a rate limiting factor for autophagy initiation, therefore regulating physiological homeostasis in different organs (16). In liver, Trp53inp2 plays a role in liver injury associated with TGF-β and BMP mediated signaling (17). More relevant to this study, Trp53inp2 has been identified through an unbiased systems genetics study using Hybrid Mouse Diversity Panel (HMDP) to be a candidate gene linking glucose utilization and cardiomyocyte hypertrophy (18). In cultured cardiomyocytes, inactivation of Trp53inp2 blunts the hypertrophic response induced by glucose and isoproterenol. However, the functional role of Trp53inp2 in intact adult heart has never been explored.

In this study, we aim at determining the physiological role of Trp53inp2 in cardiac pathological remodeling in intact heart. We established a cardiomyocyte-specific Trp53inp2 knockout mouse model (Trp53inp2-cKO) and challenged the Trp53inp2-cKO mice with severe mechanical stress (transverse aortic constriction) using 28 g needle, or moderate mechanical plus metabolic stresses using 25 g needle. Using echocardiography, molecular and histology analysis, we have uncovered that Trp53inp2 plays a protective role in heart failure induced by pressure-overload but appears to be detrimental in heart failure triggered by simultaneous application of mechanical and metabolic stresses. Further, we have provided evidence that this dual function of Trp53inp2 in cardiac pathologies is associated with its differential impact on glucose utilization.

## Materials and methods

### Animals

**Figure 1:**
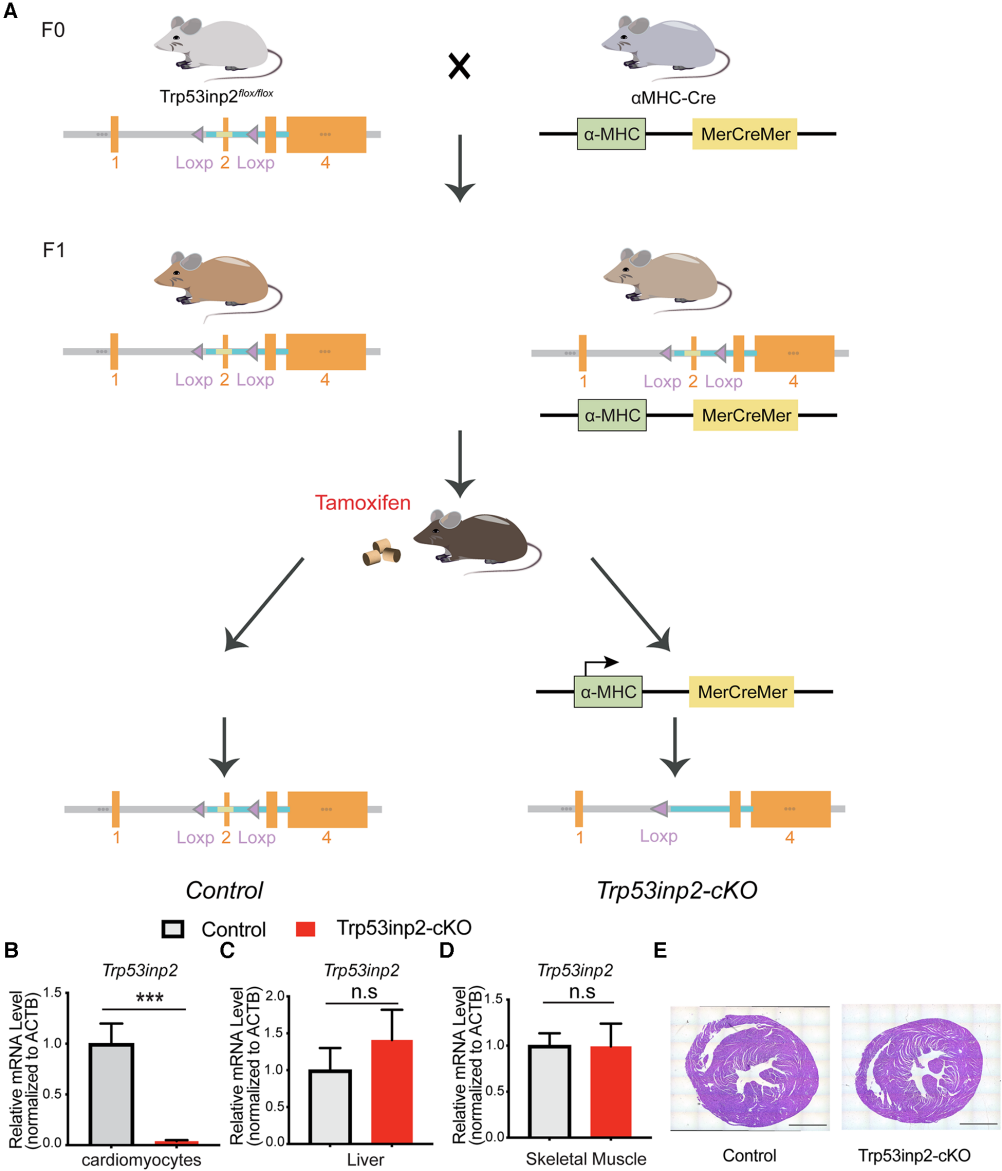
Generation of Trp53inp2-cKO mouse model. (**A**) Schematic view of Trp53inp2-cKO mouse line generation. (**B–D**) Real-time PCR analysis of Trp53inp2 expression in cardiomyocytes (**B**), liver (**C**) and skeletal muscle (**D**) between control and Trp53inp2-cKO mice. *n* = 3 each group, ***, *p *< 0.005, Student *t*-test was used for statistical analysis (**E)** H&E staining for control and Trp53inp2-cKO mouse heart at baseline. Scale bar represents 1 mm.

The mTrp53inp2 floxed allele was generated at UCSD Transgenic Mouse shared resource core (https://moorescancercenter.ucsd.edu/research/shared-resources/transgenic-mouse/index.html) via Cas9-Crispr mediated flox sites insertion flanking the exon 2 of mouse trp53inp2 gene on Chromosome 2. The *trp53inp2^flox/flox/cre^* mice (Trp53inp2-cKO) were generated by crossing the *trp53inp2^flox/flox^* mice with αMHC-Mer-Cre-Mer mice (19) where the Trp53inp2 can be inactivated in cardiomyocytes upon tamoxifen treatment as previously described (Figure 1). The genotype was confirmed by genotyping PCR for 2 flox sites. Genotyping primers: 5LoxP F: TTATGATGAT GGTAATAAGA; 5LoxP R: GGATATGGAAGAAGTGATAT; 3LoxP F: TATTTGGATGTTGGAGTCC 3LoxP R: CTAGGTGTCCCAGGTGTGTG. The genetic background of both strains is C57BL/6J. The mice were treated by Tamoxifen diet (Envigo TD 130855) for 2 weeks followed by a wash out period of 7 days. All animals in this study were handled in accordance with the Guide for the Care and Use of Laboratory Animals published by the US National Institutes of Health.

### Surgery and echocardiography

Transverse aortic constriction (TAC): The mice were placed on a volume ventilator (80 breaths/min, 1.2 ml/g/min) and anesthetized by isoflurane. The chest was opened, and the aorta was identified at the T8 region. For TAC only surgery, the suture was passed around the transverse aorta and tightened against a 28 g needle. For TAC + HFD surgery, the suture was passed around the transverse aorta and tightened against a 25 g needle.

Echocardiography: Mice were anesthetized and maintained with 2% isoflurane in 95% oxygen. A Vevo 3000 (Visual Sonics) echocardiography systems with 30 mHz scan head was used to image the heart. Both long axis and short axis view were recorded. The ejection fraction and fraction shortening were generated based on M-Mode images.

### Cardiac histological analysis

Mice were euthanized and the cardiac tissues were fixed with 10% Formalin before paraffin embedding. The H&E staining was performed at CCHMC pathology research core. For Wheat germ agglutinin (WGA) staining, cardiac tissue was cryo-preserved. Tissue sections were incubated with pre-chilled methanol at −20 °C for 10 min before blocking with 10% goat serum in 1%BSA/PBS for 1 h Alex Fluor 498 conjugated WGA (5 mg/ml, Invitrogen) were diluted in 1% BSA/PBS and incubated with section for 1hr at room temperature. Samples were counterstained and mounted with SlowFade Gold Antifade reagent with DAPI (Invitrogen). The images were taken using Leica Stellaris 8 Confocal and the cross-sectional area was quantified using Image J.

### Hexokinase activity assay

Hexokinase activity in cardiac tissues was measured using Hexokinase Activity Assay Kit (Abcam ab211103) according to the manufacturer's instruction. Briefly, ∼10 mg of left ventricle tissues were homogenized in cold assay buffer and centrifuged to remove insoluble materials. The supernatant was collected and proceed with the assay. The fluorescence was measured at Ex/Em = 535/587 in microplate reader (Biotek Synergy HT Microplate Reader) in kinetic mode for 10–30 min at 25 °C. The HK activity was calculated based on HK activity = (*B*/Δ*T* × *V*) × *D* and normalized to the original tissue weight.

### RNA extraction and quantitative PCR

Total RNA was extracted from tissues using Trizol Reagent (Thermo Fisher Scientific). RNA was used for first-strand complementary DNA synthesis using Random Primer (Thermo Fisher Scientific) and Maxima Reverse Transcriptase (Thermo Fisher Scientific) according to manufacturer's instruction. Real-time polymerase chain reaction (PCR) was performed using iTAQ SYBR Green Supermix (Bio-Rad Laboratories) with CFX Opus 96 Real-time PCR Detection System (Bio-Rad Laboratories). Values were normalized to GAPDH or ACTB. Primers are listed in Table 1.

**Table 1:** List of primers.

Primer name	Sequence
Trp53inp2 RT-F	ATGAAGTGGATGGCTGGCTC
Trp53inp2 RT-R	CTGCCGGTGACATAAACGGA
mGAPDH RT-F	ACCCAGAAGACTGTGGATGG
mGAPDH RT-R	CACATTGGGGGTAGGAACAC
mACTB RT-F	TGGCACCACACCTTCTACAA
mACTB RT-R	GTCTCCGGAGTCCATCACAA
mANF RT-F	TGAAAAGCAAACTGAGGGCT
mANF RT-R	CAGAGTGGGAGAGGCAAGAC
mBNP RT-F	GAAGGTGCTGTCCCAGATGATT
mBNP RT-R	GCTCTGGAGACTGGCTAGGACTT
mHK-1 RT-F	GCGGGTCTTCCTTTCGAATC
mHK-1 RT-R	TGAAGTCTCCGAGGCATTCA
mHK-2 RT-F	GAGGGTGGAGATGGAGAACC
mHK-2 RT-R	CAGGGGAACGAGAAGGTGAA
mCpt1a RT-F	GAAGAACATCGTGAGTGGCG
mCpt1a RT-R	AGACGTCTGGAAGCTGTACA
mPfkm RT-F	TGGCACAGTGATTGGAAGTG
mPfkm RT-R	TGGACTTTGTAGCCTCCTCG
mBDNF RT-F	GGCGCCCATGAAAGAAGTAA
mBDNF RT-R	ATGGTTTTCTTCGTTGGGCC

### Statistics

Values are expressed as mean ± SEM. Student's *t*-test, 1- or 2-way ANOVA are used to determine significant differences. *p *< 0.05 was considered as statistically significant.

## Results

### Generation of Trp53inp2-cKO mouse model

In order to understand the functional role of Trp53inp2 in intact heart physiology, we utilized Cas9-Crispr mediated gene editing to insert 2 loxP sites flanking the exon 2 of the mouse *trp53inp2* gene on Chromosome 2 (Trp53inp2^flox/flox^) (Figure 1A). To inactivate the Trp53inp2 in a cardiomyocytes specific manner, we crossed the Trp53inp2^flox/flox^ mice with αMHC-Mer-Cre-Mer mice to generate Trp53inp2^flox/flox/cre^ as well as their littermate controls Trp53inp2^flox/flox^. Both cohorts were treated with a tamoxifen diet at the age of 8 weeks for 2 weeks prior to experiments (Figure 1A). To confirm that Trp53inp2 is completely inactivated in cardiomyocytes, we isolated cardiomyocytes from Trp53inp2^flox/flox/cre^ mouse hearts and compared with the control (Trp53inp2^flox/flox^) mice for Trp53inp2 expression. Real-time PCR measurements confirmed that more than 90% knockout efficiency of Trp53inp2 was achieved in the Trp53inp2-cKO cardiomyocytes, while the expression of Trp53inp2 in liver or skeletal muscle was not affected (Figures 1B–D). Inactivation of Trp53inp2 in intact heart does not change the cardiac morphology comparing to the control littermates, as reflected by cardiac section H&E staining (Figure 1E).

### Inactivation of Trp53inp2 in intact heart accelerated pressure overload induced heart failure with reduced ejection fraction (HFrEF)

**Figure 2:**
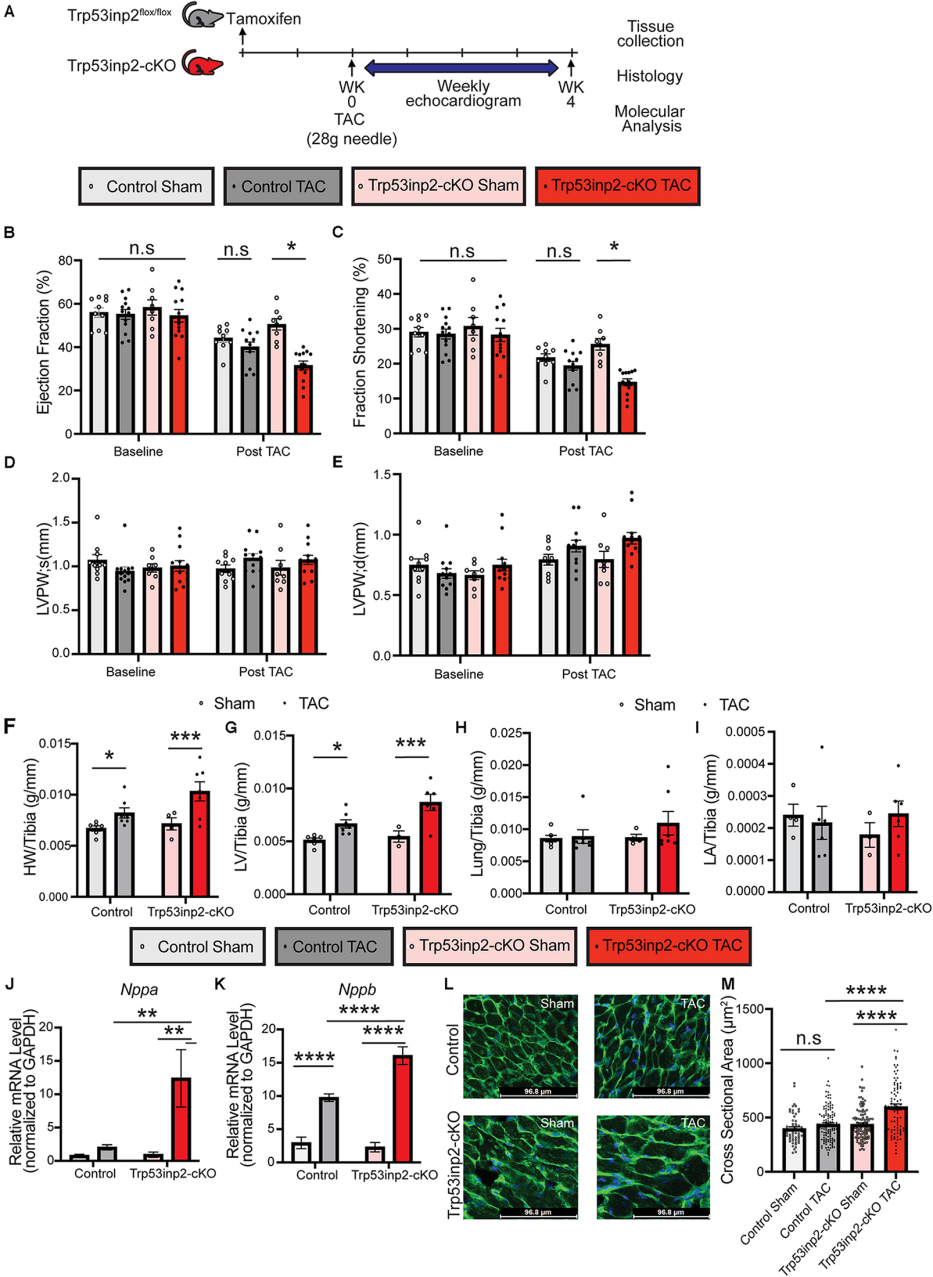
Inactivation of Trp53inp2 in heart accelerated pressure-overload induced heart failure. (**A**) Schematic view of experimental design. Control and Trp53inp2-cKO mice were subjected to sham or transverse aortic constriction using 28 g size needle (**B–E**). Echocardiography analysis including Ejection Fraction (**B**), Fraction Shortening (**C**), LVPW;s (**D**) and LVPW;d (**E**) for control and Trp53inp2-cKO mice at baseline and 4 weeks post TAC surgery. *, *p *< 0.05, Two-way ANOVA followed by Tukey's multiple comparisons test were used for statistical analysis (**F,G**). Cardiac hypertrophy status reflected by heart weight/Tibia ratio (**F**) and left ventricle weight/Tibia ratio (**G**) in control and Trp53inp2-cKO mice post TAC surgery. *, *p *< 0.05, ***, *p *< 0.005 One-way ANOVA followed by Sidak's multiple comparisons test were used for statistical analysis (**H**) Lung/Tibia ratio in Control and Trp53inp2-cKO mice post TAC surgery. One-way ANOVA followed by Sidak's multiple comparisons test were used for statistical analysis (**I**). Left atrial weight/Tibia ratio in Control and Trp53inp2-cKO mice post TAC surgery. One-way ANOVA followed by Sidak's multiple comparisons test were used for statistical analysis. (**J,K**) Real-time PCR analysis of ANF (**J**) and BNP (**K**) in control and Trp53inp2-cKO mice left ventricle tissue post-surgery. *n* = 3–5 each sample. ** *p *< 0.01, ****, *p *< 0.001 One-way ANOVA followed by Fisher's Least Significant Difference multiple comparisons test were used for statistical analysis (**L**). WGA staining of cardiac section in control and Trp53inp2-cKO mice post sham or TAC operation. (**M**) Cardiomyocytes cross section area quantification based on WGA staining. ****, *p *< 0.001, One-way ANOVA followed by Tukey's multiple comparisons test were used for statistical analysis.

Although Trp53inp2 has been suggested to play a role in glucose mediated regulation of cardiomyocytes hypertrophy *in vitro*, the *in vivo* function of Trp53inp2 has yet to be explored. We first investigated the functional role of Trp53inp2 in a severe pressure overload induced heart failure model by applying transverse aortic constriction (TAC) surgery using 28 g size needle (Figure 2A). We measured the cardiac function in the control and the Trp53inp2-cKO mice at baseline (one week post tamoxifen washout) and observed no differences in echo parameters between the control and the Trp53inp2-cKO mice (Figures 2B–E). However, at 4 weeks post TAC, the Trp53inp2-cKO mice demonstrated marked heart failure with significantly reduced ejection fraction (Figure 2B) and fraction shortening (Figure 2C) while the control mice only showed a trend in reduced cardiac function at more modest scale. Although no significant differences in ventricular wall thicknesses were observed based on echocardiography imaging between the two genotypes (LVPW; s and LVPW;d), the Trp53inp2-cKO hearts demonstrated a trend in wall thickening associated with cardiac hypertrophy (Figures 2D–E). This was supported by histological analysis at the tissue level. The Trp53inp2-cKO mice showed significant enlargement in heart and left ventricle weights (Figures 2F,G). No obvious pulmonary congestion or change of atrial sizes were observed based on lung weight and left atrial weight normalized by tibia length (Figures 2H,I). At molecular level, we observed higher levels of expression of heart failure marker genes including ANF and BNP in the Trp53inp2-cKO hearts comparing to the controls following TAC (Figures 2J,K). Lastly, enhanced cardiomyocyte hypertrophy was observed in the Trp53inp2-cKO heart following pressure overload, based on enlarged cardiomyocytes cross-section areas (Figures 2L,M). These evidence support that cardiomyocyte Trp53inp2 expression is protective and its inactivation promotes pressure overload induced pathological hypertrophic and cardiac dysfunction in intact heart.

### Inactivation of Trp53inp2 in cardiomyocytes alleviated cardiac diastolic dysfunction in response to simultaneous mechanical and metabolic stresses (cardiometabolic disorder)

**Figure 3:**
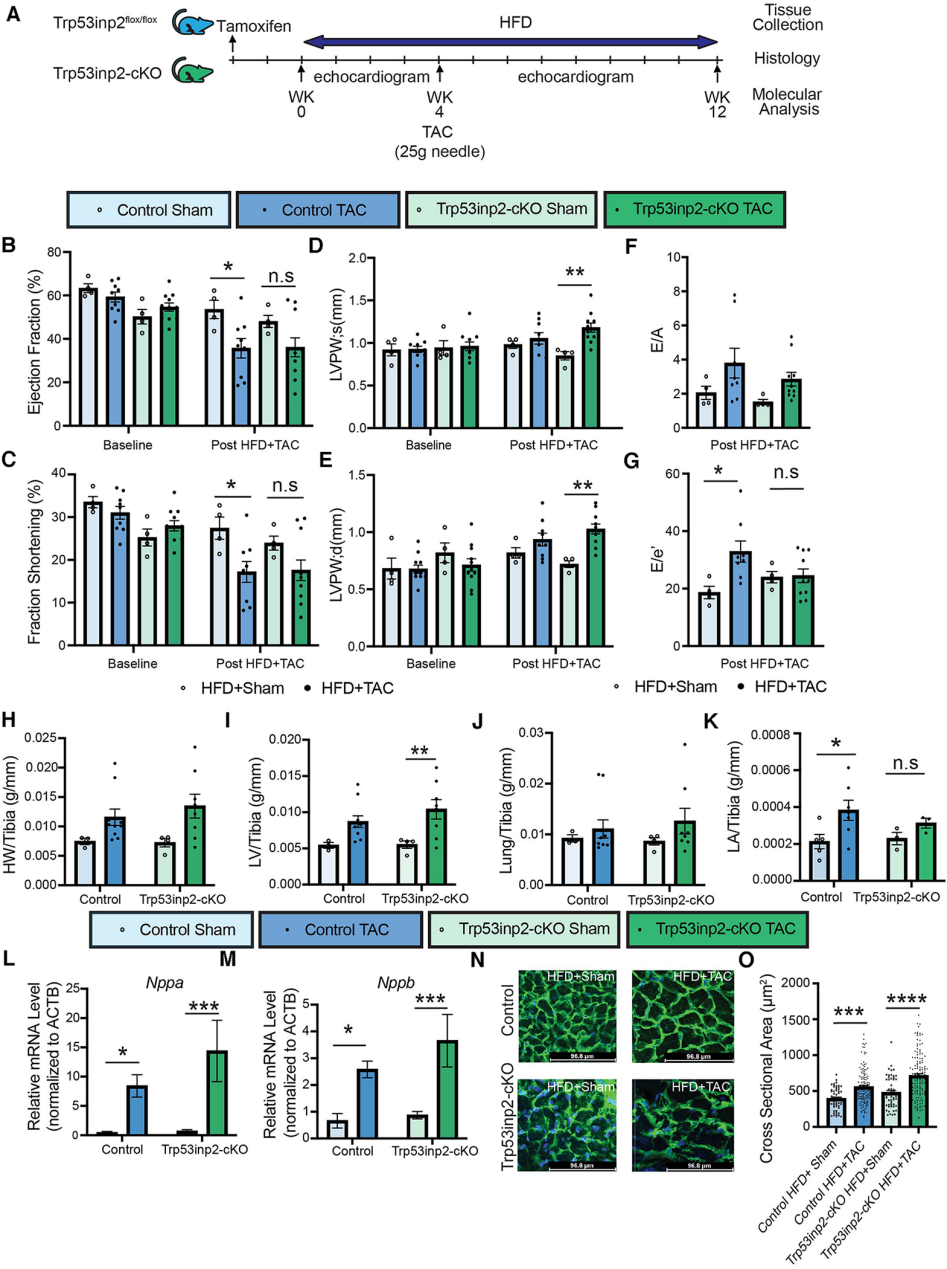
Inactivation of Trp53inp2 in heart alleviated diastolic dysfunction post cardiac and metabolic stresses (cardiometabolic disorder). (**A**) Schematic view of experimental design. Control and Trp53inp2-cKO mice were challenged with high fat diet for a total of 4 weeks before subjecting to moderate transverse aortic constriction using 25 g size needle (**B–G)**. Echocardiography analysis of Ejection Fraction (**B**), Fraction Shortening (**C**), LVPW;s (**D**), LVPW;d (**E**), *E*/*A* ratio (**F**) and *E*/*e*′ ratio (**G**) in control and Trp53inp2-cKO mice at baseline and post HFD + TAC challenge. *, *p *< 0.05, **, *p *< 0.01 Two-way ANOVA followed by Tukey's multiple comparisons test were used for statistical analysis for ejection fraction, fraction shortening, LVPW; s and LVPW; d, one-way ANOVA followed by Tukey's multiple comparisons test were used for statistical analysis for *E*/*A* and *E*′*e*. (**H,I)** Cardiac hypertrophy status measured by heart weight/Tibia ratio (**H**) and left ventricle weight/Tibia ratio (**I**) in control and Trp53inp2-cKO animals post sham or TAC operation. ** *p *< 0.01 One-way ANOVA followed by Sidak's multiple comparisons test were used for statistical analysis (**J**). Lung/tibia ratio in control and Trp53inp2-cKO mice post sham or TAC operation. One-way ANOVA followed by Sidak's multiple comparisons test were used for statistical analysis (**K**). Left atrial weight/Tibia ratio in Control and Trp53inp2-cKO mice post TAC surgery. One-way ANOVA followed by Uncorrected Fisher's LSD multiple comparisons test were used for statistical analysis, *, *p* < 0.05 (**L,M**) Real-time PCR analysis of ANF (**L**) and BNP (**M**) in control and Trp53inp2-cKO mice left ventricle tissue post-surgery. *n* = 4–5 each sample. *, *p *< 0.05; ***, *p *< 0.005 One-way ANOVA followed by Fisher's Least Significant Difference multiple comparisons test were used for statistical analysis (**N**). WGA staining of cardiac section in control and Trp53inp2-cKO mice post sham or TAC operation (**O**). Cardiomyocytes cross section area quantification based on WGA staining. ***, *p *< 0.005, ****, *p *< 0.001. One-way ANOVA followed by Tukey's multiple comparisons test were used for statistical analysis.

Next, we explored the role of Trp53inp2 in the pathogenesis of heart failure induced by a combined stresses of moderate mechanical stress and metabolic overload. We challenged the Trp53inp2-cKO mice and their age-matched littermate controls with continuing high fat diet (HFD). At 4 weeks post HFD, additional mechanic stress was induced by applying a moderate pressure overload using TAC with a 25 g needle (Figure 3A). The combination of the moderate pressure overload and metabolic stress would lead to cardiometabolic disorder, characterized by both systolic and diastolic dysfunction. Cardiac function was monitored for additional 8 weeks following TAC. In contrast with the results observed in the pressure overloaded lean mice, pressure-overload in the HFD treated control mice led to significantly reduced ejection fraction (Figure 3B) and fraction shortening (Figure 3C). On the other hand, the statistic significances for the reduction of ejection fraction and fraction shortening were diminished in the Trp53inp2-cKO mice (Figures 3B,C). Importantly, the diastolic dysfunction observed in the control mice was significantly attenuated in the Trp53inp2-cKO mice (Figures 3F,G). These changes in cardiac function appeared to be independent from the status of hypertrophy since the cardiac hypertrophy remained significantly induced in the Trp53inp2-cKO heart at comparable level as the controls based on echo parameters such as LVPW; s (Figure 3D) and LVPW;d (Figure 3E), tissue weights, marker gene expression and cross-section area measurements (Figures 3H–O). Remarkably, left atrial sizes showed a significant induction in control mice, but not in Trp53inp2-cKO mice, further supporting our conclusion that Trp53inp2 plays a role in cardiac diastolic function in cardiometabolic disease (Figure 3K). In summary, combined cardiac and metabolic stresses led to both diastolic and systolic dysfunction in control mice, however, inactivation of Trp53inp2 in cardiomyocytes protected the heart from both systolic and diastolic dysfunction without an impact on cardiac hypertrophic remodeling.

### Trp53inp2 serves as a molecular switch for glucose utilization under different cardiac stresses

**Figure 4:**
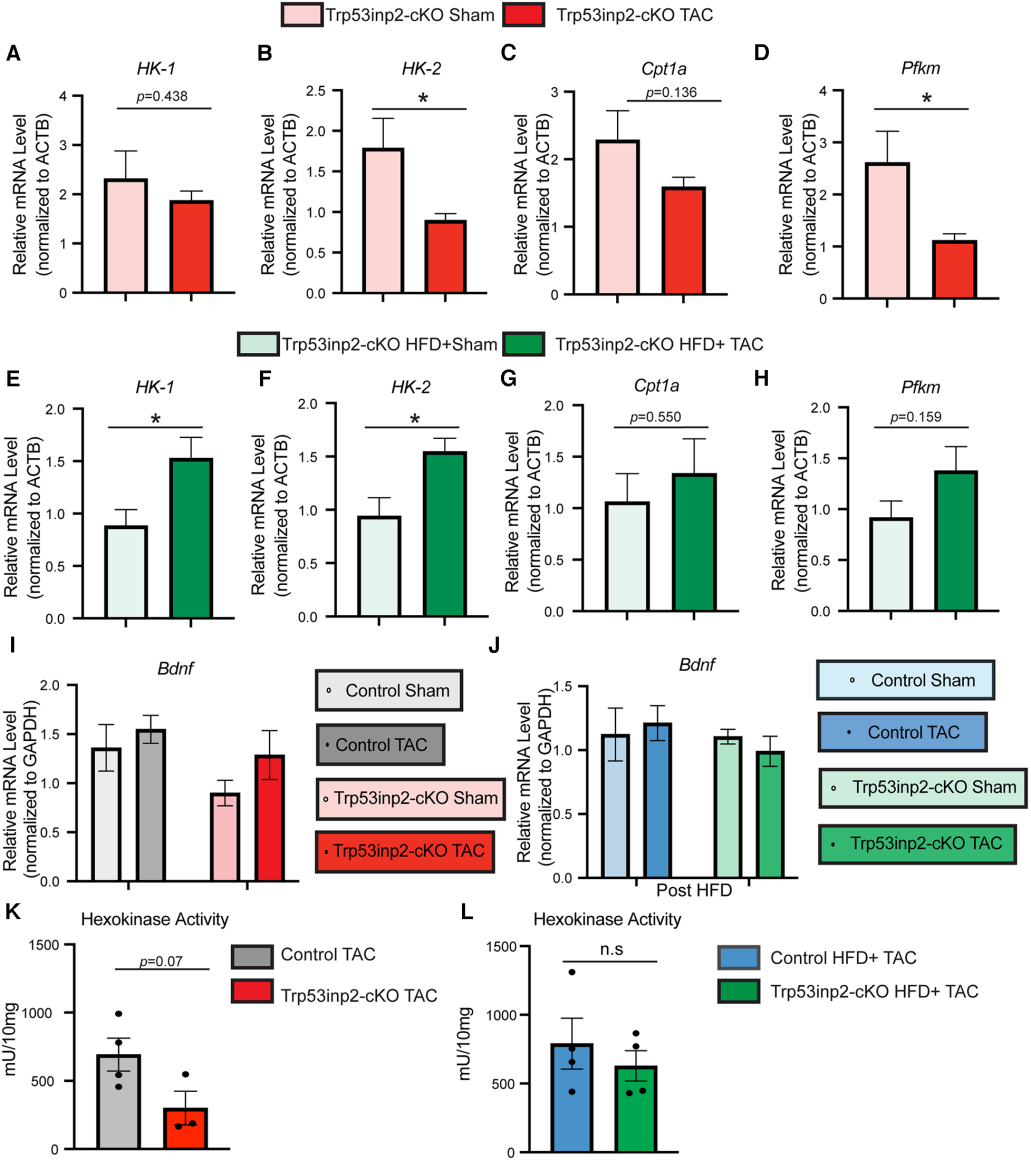
Trp53inp2 plays an important role in glucose utilization under different cardiac stresses. (**A–D)** Real-time PCR analysis of HK-1 (**A**), HK-2 (**B**), Cpt1a (**C**) and Pfkm (**D**) in Trp53inp2-cKO mice left ventricle tissue post sham or TAC surgery. *n* = 3–5 each group, *, *p *< 0.05 Student *t*-test was used for statistical analysis. (**E–H)** Real-time PCR analysis of HK-1 (**E**), HK-2 (**F**), Cpt1a (**G**) and Pfkm (**H**) in Trp53inp2-cKO mice left ventricle tissue post sham or TAC surgery with HFD. *n* = 4–5 each group, *, *p *< 0.05, Student *t*-test was used for statistical analysis. (**I-J)** Real-time PCR analysis of BDNF in control and Trp53inp2-cKO mice left ventricle tissue post pressure overload injury **(I**) or post cardiometabolic stress (**J**). *n* = 4–5 each group. Two-way ANOVA followed by Fisher's test were used. (**K,L)** Hexokinase activity in control TAC and Trp53inp2-cKO TAC hearts (**K**) or control HFD + TAC comparing with Trp53inp2-cKO HFD + TAC hearts (**L**). Student *t*-test was used for statistical analysis.

Previously, Trp53inp2 has been suggested to play an important role in cardiomyocytes glucose utilization linking with hypertrophy based on *in vitro* analysis (18). The different outcome of Trp53inp2 inactivation under different stresses raised the question about its impact on glucose metabolism. Therefore, we performed real-time PCR to determine the expression of selected glucose utilization genes in the same mouse hearts obtained from this study. Strikingly, a diametrically different expression pattern of glucose utilization genes was observed in different stress conditions. Under single pressure overload, inactivation of Trp53inp2 in cardiomyocytes led to a significant reduction of glucose utilization genes including HK-2 and Pfkm (Figures 4A–D). In sharp contrast, the Trp53inp2-cKO mouse hearts showed a significant induction of these glucose utilization genes including HK-1 and HK-2 in cardiometabolic disorder (Figures 4E–H). Therefore, the different functional impact on cardiac contractile function from Trp53inp2 inactivation may be highly correlated with its impact on glucose utilization. However, the impact of Trp53inp2 KO on cardiac hypertrophy does not appear to be related to glucose utilization as originally predicted. To exclude the cardiac amyloidosis as a potential cause for the cardiac hypertrophy that we observed in our Trp53inp2-cKO mice, we further evaluated BDNF, a marker for cardiac amyloidosis (20). Real-time PCR analysis showed no significant changes of BDNF in either our pressure overload model or cardiometabolic disorder model, providing support for our conclusion that Trp53inp2 impacts on cardiac function through glucose utilization and that hypertrophic response we have observed in our study was bona fide pathological remodeling to pressure overload (Figures 4I,J). Lastly, to understand whether inactivation of Trp53inp2 in intact heart directly impacts on the hexokinase enzymatic activity, we performed hexokinase activity assay using the cardiac tissue from the two disease models. Interestingly, inactivation of Trp53inp2 in heart led to a significant decrease of hexokinase activity in response to severe pressure overload (Figure 4K), the hexokinase activity in Trp53inp2-cKO hearts showed a similar trend with control littermates in our cardiometabolic disorder model (Figure 4L). This data suggested that the changes in hexokinase activity could partially explain the differential cardiac phenotype we observed in Trp53inp2-cKO mouse. Additional analysis is necessary to understand the protection in diastolic function in Trp53inp2-cKO mice post metabolic challenge.

In summary, we have uncovered for the first time the *in vivo* function of Trp53inp2 in cardiac hypertrophy and dysfunction through a cardiomyocyte specific Trp53inp2 knockout mouse model. We find the impact of Trp53inp2 on cardiac contractility is stress-dependent and highly correlated with its impact on glucose utilization related genes. On the other hand, the impact on hypertrophy is not stress-specific or correlated with glucose utilization. These insights suggest that Trp53inp2 is a potentially important molecular link between glucose utilization and cardiac contractility and can have a protective or detrimental role depending on the nature of pathological stress.

## Discussion

Our study demonstrated functional role of Trp53inp2 in cardiac pathological remodeling in intact heart. Inactivation of Trp53inp2 in cardiomyocytes led to distinct outcome in response to different cardiac diseases. In severe pressure overload induced heart failure, inactivation of Trp53inp2 has detrimental effect accelerating the heart failure progression characterized by significantly reduced ejection fraction. In response to combined metabolic and moderate cardiac stresses, inactivation of Trp53inp2, however, protected heart from cardiac systolic and diastolic dysfunction post injury.

The function of Trp53inp2 has been characterized in liver as well as skeletal muscle (14). In cultured cardiomyocytes, inactivation of Trp53inp2 suppresses expression of key glycolytic enzymes. Inactivation of Trp53inp2 further blunts glucose and isoproterenol treatment induced cardiac hypertrophy *in vitro* (18). Our *in vivo* model provides strong supportive evidence that Trp53inp2 protects the heart from combined metabolic and cardiac stresses based on improved cardiac systolic and diastolic function (a decrease in ejection fraction and fraction shortening in control but not Trp53inp2-cKO mice post TAC + HFD; and an increase in *E*/*e*′ in control but not Trp53inp2-cKO mice post TAC + HFD). However, our *in vivo* mouse model further showed distinct outcome when the Trp53inp2-cKO mice are challenged with transverse aortic constriction surgery alone, leading to accelerated heart failure. One possible explanation is the complexity of the *in vivo* system including the organ-organ crosstalk as well as interaction among different cell types within the myocardium. As the cardiometabolic disorder essentially is a multi-organ dysfunction model, the crosstalk between the adipose tissue and liver with the heart post combined cardiac and metabolic stresses could explain the different roles of Trp53inp2 in different cardiac diseases. A global change of glucose homeostasis and insulin resistance is possible in the Trp53inp2-cKO mice post high fat diet and cardiac injury and a complete metabolic profiling, as well as analysis of involvement of mTOR signaling pathway among different tissues would better provide the underlying mechanism for the physiological role of Trp53inp2 in heart.

The Trp53inp2 was originally discovered as a transcription factor with the capacity of binding on promoter of thyroid hormone receptor-α. Further studies have also identified its regulatory role in autophagy in both flies and mammals (21–23). Although the changes we observed in glucose metabolic genes expression in Trp53inp2-cKO mouse ventricular tissues could provide partial explanation for the physiological responses in these animals, several important questions need to be addressed. Firstly, the underlying mechanism for Trp53inp2 mediated glucose metabolic gene expression. As Trp53inp2 has been first identified to be a transcription activator, it is possible that the Trp53inp2 directly bind to the promoter region of these glucose metabolic genes. This is unlikely the case as we have observed different change directions in the Trp53inp2-cKO mouse hearts in response to different cardiac stresses. Secondly, the changes of glucose metabolic genes could be directly contributing to the physiological impact of Trp53inp2 in heart, or they could be merely secondary effect due to the protected cardiac function. The same concern applies to the hexokinase activity analysis post different stresses. A combined gene manipulation could help resolve this issue and an unbiased gene discovery approach would also yield further insights on the cardiac remodeling in these mice.

Our study provided interesting concept that one protein regulator could play dual roles in response to different cardiac stresses. With the cardiometabolic syndrome being the largest unmet medical need in the past decade, additional research in the dual-role regulators would for sure yield promising therapeutic strategies for this disease in the very near future.

## Data Availability

The original contributions presented in the study are included in the article/Supplementary Material, further inquiries can be directed to the corresponding author.
